# Dengue haemorrhagic fever among adults – An observational study in Chennai, south India

**Published:** 2010-12

**Authors:** M. Emmanuel Bhaskar, Swathy Moorthy, N. Senthil Kumar, Preetam Arthur

**Affiliations:** Department of Medicine, Sri Ramachandra Medical College & Research Institute Sri Ramachandra University, Chennai 600 116, India

Sir,

The spectrum of disease due to dengue infection ranges from a subclinical or mild illness to a severe form of haemorrhagic fever which may prove fatal^1^. Dengue haemorrhagic fever (DHF) is prevalent among individuals of all age in north India. Though uncomplicated dengue fever (DF) is prevalent among children and adults in Chennai, DHF has been largely restricted to infants and children^2^. Since 2005 we have been witnessing DHF among young as well as elderly adults in Chennai, Tamil Nadu, India. This study was initiated to systematically observe the clinical presentation and factors associated with mortality due to DHF among adults aged more than 18 yr in Chennai, south India.

The study included consecutive adult patients (aged > 18 yr) who were admitted to Sri Ramachandra Medical College and Research Institute, a tertiary care centre in Chennai between August 2007 and February 2009 (two consecutive monsoon seasons). Patients were required to fulfill the 1997 World Health Organization (WHO) case definitions for DHF on admission or during hospital stay^1^. Patients with dengue fever who lack DHF case definition were not included in the study. DHF and its grade (I to IV) was defined as per WHO guidelines^1^.

Patients were interviewed and examined with an objective of identifying the symptoms and signs associated with current febrile illness, associated co-morbidities and other baseline characteristics. This was followed up with basic blood tests (biochemical and haematological), urine analysis, chest X-ray and ultrasound abdomen. IgM and IgG antibodies against dengue was detected using a commercial enzyme-linked immunoassay kit (Panbio, Brisbane, Australia). Additional tests like IgM antibodies for leptospirosis (Panbio, Brisbane, Australia), smear for malaria, blood culture, urine culture, d-dimer, partial thromboplastin time, endoscopy, *etc*., were done on clinical suspicion. Informed written consent was obtained from all stable patients and from the closest relative of patients who were critically ill. The ethics committee of Sri Ramachandra University approved the study protocol.

Continuous variables were expressed as mean ± SD and categorical variables were expressed as number (%). Association between patient factors (age, sex, co-morbidities, gastrointestinal bleeding, haematocrit, platelet count, albumin, prothrombin time, renal failure, shock and dual infection) and mortality was studied using chi-square test or Fisher exact test for categorical variables and Mann-Whitney U test for continuous variables. *P*<0.05 was considered to be statistically significant.

A total of 128 patients were admitted with features of DHF during the study period; 109 (85%) were admitted during monsoon seasons. The mean age was 33 ± 15 yr; 15 (11.7%) were older than 60 yr. Overall, the proportion of male and female were equal (1:1). A small proportion of patients had co-morbidities like diabetes 5 (3.9%), hypertension 5 (3.9%) or chronic obstructive pulmonary disease (COPD) 2 (1.5%) and none of them had pre-existing chronic kidney disease. Mean duration of fever was 4 days (range 2-6 days). The most frequent symptom was fever 128 (100%) followed by myalgia 88 (68%), gingival bleed 81 (63%), headache 73 (57%), fatigue 54 (42%) and arthralgia 26 (20%). Fifty nine patients (46%) had petechiae or purpura making it the most frequent clinical sign in our study population followed by altered sensorium 24 (19%), sub-conjuctival bleed 15 (12%), ecchymosis 14 (11%), rash 13 (10%), malena 12 (9%) and menorrhagia 11 (8%) : 28 (22%) had hepatomegaly and 6 (5%) had splenomegaly. Also, 47 (37%) had hypotension defined as systolic blood pressure less than 90 mm Hg.

The mean duration between onset of fever and sampling for initial investigation was 5 days (range 3-9 days). Seventy three (57%) had leucopenia defined as total count (TC) < 4000 per µl; 23 (18%) had a platelet count of less than 20, 000 per µl and none had a platelet count of less than 10,000 per µl. Evidence of hepatitis defined as transaminase elevation ≥ 400 IU/l was seen in 35 (27.3%) (Table). Of them, 24 (18.6%) had a probable ischaemic hepatitis since transaminase values were > 1000 IU/l, they were in shock and their transaminases showed rapid resolution within 72 h. Eighty patients (62.5%) had pleural effusion by ultrasound compared to 35 (27%) by chest X-ray; 14 had ultrasound evidence of acalculous cholecystitis (gall bladder wall thickening) of whom only two had abdominal pain and tenderness. IgM antibodies against dengue was positive in 59 (46%) cases, of whom 20 (15.6%) had additional IgG antibodies. Co-existent serology proven leptospirosis was present in 8 (6.2%) and microscopy proven falciparum malaria in 6 (4.6%). None had triple infection. Blood culture drawn on admission were sterile in all patients.

**Table:** Haematological & biochemical parameters

Variable	DHF I* n=4	DHF II* n=52	DHF III* n=50	DHF IV* n=22	Overall† n=128
Hb% (mg/dl)	16.4	14.8	13.3	17	14.6 ± 3.2
PCV (%)	48	43	39	45	42 ± 9
TC (cells/µl)	3450	3870	4373	3706	4025 ± 2028
Platelet count (cells/µl)	40500	46384	50840	20245	43448 ± 24048
Creatinine (mg/dl)	0.7	1.1	0.8	2.7	1.2 ± 0.8
BUN (mg/dl)	10	19	14	51	22 ± 17
T. Bilirubin (mg/dl)	1.2	1.3	1.3	2.6	1.5 ± 1
D. Bilirubin (mg/dl)	0.6	0.6	0.5	1.3	0.7 ± 0.6
ALT (IU/l)	295	255	268	737	344 ± 350
AST (IU/l)	258	225	226	649	299 ± 314
SAP (IU/l)	89	117	121	178	128 ± 53
Albumin (g/dl)	3.2	3.2	3.1	2.8	3.1 ± 0.6
Globulin(g/dl)	2.9	3.3	3.1	3.3	3.2 ± 0.4
INR	1.5	1.4	1.5	2	1.6 ± 0.4

TC, total count; PCV, packed cell volume; BUN, blood urea nitrogen; ALT, alanine transaminase; AST, aspartate transaminase; SAP, serum alkaline phosphatase; INR, International Normalized Ratio

* variable expressed as mean

† variable expressed as mean ± SD

Six of 11 patients who had symptoms of upper gastrointestinal bleed underwent endoscopy which showed fundal erosions (n=3), duodenal ulcer (n=1) and duodenitis (n=2). Ten patients required ventilatory support within the first 48 h of admission due to impaired mentation of whom five continued to receive mechanical ventilator support till death. Ventilator associated pneumonia occurred in two and acute respiratory distress syndrome (ARDS) complicated the course in one of them. Disseminated intravascular coagulation (DIC) was present in 4 patients. Platelet concentrates were administered to 68 (53%). 34 (26%) received fresh frozen plasma. 66 (51%) received antibiotics and 20 (15.6%) received antibiotics along with artesunate. Refractory shock was present in 16 (12.5%) and progressive renal failure occurred in 15 (12%). The mortality rate was 14 per cent (18 of 128).

Level of creatinine on admission of 2 mg/dl or higher (*P*< 0.001), progressive renal failure (*P*< 0.001), refractory shock (*P*< 0.001), dual infection (*P*< 0.001), admission platelet count < 20000 per µl (*P*=0.009) and admission INR>2 (*P*=0.02) were associated with mortality. While age, sex, presence of gastrointestinal bleeding, presence of co-morbidities, higher PCV at admission and serum albumin at admission <2.5 mg/dl, were not associated with mortality.

Though uncomplicated dengue fever occurs both in adults and children in Chennai, till recently DHF in Chennai has been predominantly restricted to children^3^–^5^. Salient differences in clinical features in our study population compared with previous studies included more cases of sub-conjuctival bleed, gum bleeding, altered sensorium and a lower occurrence of rash, arthralgia and gastrointestinal symptoms^6^–^9^. The low occurrence of DHF grade-I in our study could be due to selection bias since only patients with severe illness were included.

Though the severity of thrombocytopenia in our study as reflected by the mean platelet count (43488/ µl) and the proportion of patients having a platelet count of less than 20000/µl (18%) was lesser compared to previous reports, the frequency of bleeding tendencies was comparable^6^,^9^,^10^.

The mean alanine transaminase (344 IU/l) observed in our study was markedly higher compared to the range of mean alanine transaminase (75 to 143 IU/l) reported in previous studies^6^,^10^. This could be due to increased occurence of ischaemic hepatitis as a complication of hypotension in our study population. The low prevalence of symptomatic acalculous cholecystitis in our study is similar to reports from North India^6^.

Mortality rate due to DHF in adults ranges from 1-8 per cent^6^,^8^,^9^. The 14 per cent mortality observed in our study was a cause for concern. Similar to our observation, acute renal failure has been consistently identified as a risk factor of mortality in patients with DHF^11^,^12^. The 2009 WHO guidelines on dengue has included organ dysfunction as an indicator of severe illness. Though the new diagnostic criteria are in field testing, these have taken into account the impact of organ dysfunction on disease outcome^13^. Dual infection has been shown to increase the mortality in DHF by about 14- fold^8^. This fear partially explains although does not justify the high antibiotic use in our study. Concurrent infection due to malaria and dengue observed in six patients in our study was relatively uncommon^14^,^15^. The fact that our patients who had severe thrombocytopenia or significant coagulopathy on admission had unfavourable outcome despite the liberal use of platelet transfusion and fresh frozen plasma indicates the importance of other factors in the outcome of DHF.

In conclusion, the DHF is spreading across all age groups in Chennai, south India. Given the high complexity of the nature of the disease and failure of containment strategies, more scientific inputs are required to decrease the morbidity and mortality in these patients.
